# Complete Genome Sequences of pLMA1 and pLMA7, Two Large Linear Plasmids of *Micrococcus* Strains Isolated from a High-Altitude Lake in Argentina

**DOI:** 10.1128/genomeA.00010-18

**Published:** 2018-02-22

**Authors:** Julian Rafael Dib, María Florencia Perez, Jörg Schuldes, Anja Poehlein, Martin Wagenknecht, María E. Farías, Friedhelm Meinhardt, Rolf Daniel

**Affiliations:** a PROIMI-CONICET, Tucumán, Argentina; b Instituto de Microbiología, Facultad de Bioquímica, Química y Farmacia, Universidad Nacional de Tucumán, Tucumán, Argentina; c Genomic and Applied Microbiology & Göttingen Genomics Laboratory, Institute of Microbiology and Genetics, Georg-August University of Göttingen, Göttingen, Germany; d Institut für Molekulare Mikrobiologie und Biotechnologie, Westfälische Wilhelms-Universität Münster, Münster, Germany

## Abstract

The two linear plasmids pLMA1 (109,112 bp) and pLMA7 (82,075 bp) from *Micrococcus* strains were isolated from a high-altitude lake in the Argentinean Puna, sequenced, and annotated. These extrachromosomal elements are probably conjugative and harbor genes potentially involved in coping with the harsh conditions in such extreme environments.

## GENOME ANNOUNCEMENT

*Micrococcus* sp. A1 and A7 were isolated from Laguna Azul, a high-altitude lake (4,600 m above sea level) in the northwest of Argentina (1). In this environment, extreme conditions prevail, such as oligotrophy, high UV radiation, high arsenic concentrations, and low oxygen pressure. *Micrococcus* sp. A1 and A7 host the large linear plasmids pLMA1 and pLMA7, respectively (2, 3). As the hosts’ adaptation traits might be, at least partially, conferred by pLMA1 and pLMA7, the plasmids were sequenced and annotated.

Here, we present the complete nucleotide sequences of the linear plasmids pLMA1 and pLMA7. The plasmids were isolated by pulsed-field gel electrophoresis and purified from gel by electroelution. The linear plasmid pLMA7 was sequenced using a Sanger sequencing approach. The isolated DNA was used to construct a plasmid library according to the TOPO TA library construction kit manual (Life Technologies, Darmstadt, Germany). In total, 768 recombinant plasmids were end sequenced with an ABI 3730xl DNA sequencer (Life Technologies, Darmstadt, Germany), processed with Phred, and assembled using Phrap (http://www.phrap.org). Sequence editing was done using GAP4 as part of the Staden software package (4), and final gap closure was performed by PCR and primer walking using a Bio-X-Act kit (Bioline, London, United Kingdom).

The linear plasmid pLMA1 was sequenced by a combination of Sanger sequencing and 454 pyrosequencing. This strategy was previously successfully applied for analysis of cloned fragments of pLMA1 (5). The 454-shotgun library was constructed and sequenced with the Genome Sequencer FLX system using titanium chemistry as recommended by the manufacturer (454 Life Sciences, Roche Applied Science, Branford, CT). Approximately 85,000 shotgun reads were generated and assembled *de novo* into eight large contigs (>500 bp) using Roche Newbler assembler software 2.0 (454 Life Science). In addition, 96 recombinant plasmids were end sequenced by Sanger sequencing and joined with pyrosequencing-derived contigs. Sequence editing was done as described above. As the plasmids’ termini are covered by terminal proteins covalently linked to the DNA 5ʹ ends in both linear plasmids, the telomeric sequences were not obtained by the sequencing protocols used. Annotation was performed by the Integrated Microbial Genomes (IMG) annotation pipeline (6).

The nucleotide sequence of pLMA1, with a G+C content of 68.4%, consists of 109,112 bp comprising 125 predicted protein-encoding genes, of which 64 were assigned to known functions. The plasmid pLMA7 comprises 82,075 bp (69.5% G+C) and harbors 93 predicted protein-encoding genes, of which 25 were assigned to known functions.

Besides plasmid backbone genes, including genes for conjugation and replication, pLMA1 and pLMA7 harbor several accessory genes related to resistance to extreme environmental conditions. In the case of pLMA1, genes involved in the hydrolytic defluorination of fluoroacetate as well as in the resistance to different chemicals were found. Checking the resistance profile of a cured pLMA1-deficient derivative of *Micrococcus* sp. A1, we obtained experimental evidence for a plasmid-encoded erythromycin resistance (2). Accordingly, analysis of the pLMA1 sequence revealed a potential erythromycin resistance gene (rRNA adenine *N*-6-methyltransferase). Interestingly, pLMA7 encodes a putative cobalt-zinc-cadmium efflux system which is potentially involved in coping with heavy-metal poisoning.

### Accession number(s).

The nucleotide sequences of the linear plasmids pLMA1 and pLMA7 have been deposited in the GenBank database under the accession numbers LK056645 and KJ599675, respectively. The versions described here are versions LK056645.1 and KJ599675.1, respectively.
